# The Relationship Between Self-Care Behavior and Concerns About Body Image in Patients Undergoing Hemodialysis in Iran

**DOI:** 10.3389/fpubh.2022.825415

**Published:** 2022-03-04

**Authors:** Hamid Sharif Nia, Daniyal Kohestani, Erika Sivarajan Froelicher, Fatima Muhammad Ibrahim, Maryam Mohammad Ibrahim, Fatemeh Bayat Shahparast, Amir Hossein Goudarzian

**Affiliations:** ^1^Psychiatry and Behavioral Sciences Research Center, Addiction Institute, Mazandaran University of Medical Sciences, Sari, Iran; ^2^Student Research Committee, School of Nursing and Midwifery, Iran University of Medical Sciences, Tehran, Iran; ^3^Department of Physiological Nursing, Schools of Nursing, University of California, San Francisco, San Francisco, CA, United States; ^4^Department of Epidemiology, Biostatistics, and Medicine, University of California, San Francisco, San Francisco, CA, United States; ^5^Reproductive Health Department, School of Nursing and Midwifery, Tehran University of Medical Sciences, International Campus, Tehran, Iran; ^6^Department of Psychiatric Nursing, Mazandaran University of Medical Sciences, Sari, Iran

**Keywords:** self-care behavior, body image concern, hemodialysis, Iran, chronic renal failure

## Abstract

**Background and Aim:**

Hemodialysis treatment saves the life of people with end-stage renal disease (ERDS), but does not prevent the suffering of the disease, anxiety, hopelessness, and so on. Many studies have been performed on self-care behaviors as well as body image in different patients, but so far, no research has been done to investigate the relationship between self-care behavior and body image concerns in hemodialysis patients. Therefore, the aims of this study are to determine the relationship between self-care behavior and body image concern in patients undergoing hemodialysis.

**Materials and Methods:**

A cross-sectional design was used to evaluate Self-care Behavior and Concerns about Body Image in a convenience sample of 280 patients with ERDS. Measures included demographic factors, health characteristics, and validated instruments of the study constructs Body Image Concern Questionnaire (BICI) and Assessment of self-care behaviors with arteriovenous fistula (ASBHD-AVF).

**Results:**

This study showed that self-care behaviors have the ability to predict body image concerns. There was a negative and significant relationship between self-care behavior and body image concern (*B* = −0.162, β = −0.140, *p* = 0.020). These variables explain 7.5% of predictors.

**Conclusion:**

Although the findings of the present study showed that increasing age and improving the level of self-care behaviors in patients undergoing hemodialysis reduces body image anxiety, but women were the strongest predictor of body image anxiety.

## Introduction

End-stage renal disease (ESRD) is a chronic and debilitating disorder; whereby a patient requires peritoneal dialysis or hemodialysis (several times a week) to survive (1). Conditions that result in ESRD in individuals include type 2 diabetes, hypertension, glomerulonephritis and interstitial nephritis (2). In the United Kingdom, over 115 new patients per million receive dialysis treatment annually. Reports from the United States suggest that the prevalence of ESRD increased by 600% between 1980 and 2009. Also, more than 1 in 7, that is 15% of US adults or 37 million people, are estimated to have CKD (3). According to reports from the Iranian management center for transplantation and special diseases, the prevalence of ESRD among the Iranian population has increased over the last few years (4). Overall, the prevalence of ESRD varies across countries (2).

Hemodialysis treatment saves the lives of people with ESRD, but it does not prevent the pain associated with the disease. The number and seriousness of symptoms increase with age and length of dialysis treatment (5). These people will be confronted with lifestyle limitations to cope with their illness, which can greatly affect people's social functioning, beliefs, sense of control, compatibility, and ultimately their body image (6). Body image is a patient's mental image of their body, which is a complex structure with thoughts, feelings, evaluations and behaviors related to the body, and it has a great impact on the formation of individual identity (7). There are different criteria for evaluating a patient 's self-image, which vary in different societies (8).

In fact, the body with which we live plenarily influences our life experiences. As Plato stated: “Like oysters, which are attached to their shells, we are also attached to our bodies”. People tend to keep their body image intact and endeavor not to be affected by changes, injuries or losses in terms of function or body organs. Because any change is considered a threat by, the individual and can lead to anxiety and fear. The most common conditions that lead to changes in body image from the perspective of individuals are receiving medical treatment, amputation, and changes in body functions. Patients with ESRD experiences several clinical conditions at the same time that can lead to a change in body image (9, 10).

Patients undergoing hemodialysis experience both physical and psychological problems such as feelings of fear, anxiety, and hopelessness. Also, patients often suffer from body image disorders. Patients with ESRD often have impaired body image and have difficulty accepting it due to changes in their physical appearance (11). Dissatisfaction with the body is accompanied by social anxiety, shame, depression, decreased self-efficacy, and reduced quality of life (12). Self-efficacy plays an important role in influencing individual behavior. Strong feelings of self-efficacy are associated with better health, greater success, and greater social integration, while feelings of low self-efficacy are associated with depression, stress, and decreased motivation and cognitive abilities (13). Self-efficacy is the main predictor of self-care behaviors (14). It is conscious self-care behaviors that enables people to consciously engage in appropriate self-care behaviors (15).

In addition, dialysis affects the life of the patient and the family, so it is necessary to have an effective strategy in order to reduce dependence, maintain self-esteem, and reduce concerns about body image of individuals. However, studies show that health care providers do not know enough about body image (16). Self-care is a viable strategy for acclimating to life events and stresses, which leads to independence and promotes health by sticking to treatment and performing routine life activities (17). Self-care is characterized as the performance of individual duties to protect life, health, and well-being, that are progressively made (18). Maintaining people's participation in self-care interventions is especially important when a patient has a chronic illness. People with ESRD can adapt to the changes needed to improve their health and take full responsibility for their caregiving activities with active participation (19).

Positive engagement with their family members can lead to better decisions and self-care. Many people need psychological support from health care groups, and it is important to recognize this at the beginning (20). When a patient complains of physical discomfort, it is important to understand that his or her body image has changed, as this will be effective in providing supportive measures (10). Orem is one of the theorists that talks about self-care. According to this theorist, one should take care of oneself and others (21). A study conducted by Cook-Cottone (15) showed that active participation in health care affects body image (15). Other researchers have also shown that self-conscious care is introduced as an antidote to social pressures as well as an enforceable set to foster positive imagery.

If people have a positive body image despite the changes in their bodies appearance, their behaviors will show more self-care, which will ultimately improve the quality of their health (15). Self-care behaviors such as medical care, nutrition, hydration, and exercise are enhanced by strengthening the inner aspects of a patient 's perception of their body. Attention to the body needs is a necessary aspect of self-care behaviors and is likely associated with the positive body image (15). According medical sciences, body image can affect the results of medical interventions, especially for chronic diseases (16). There are many studies on self-care behaviors and body image in different patients (22–25), but no research has been done to investigate the relationship between self-care behavior and body image concerns in hemodialysis patients. Thus, the present study aims to determine the relationship between these two variables in patients undergoing hemodialysis.

## Materials and Methods

### Design and Participants

This study uses a cross sectional study design. It was conducted in 2018. The sample size was estimated using by G^*^Power 3.1.7 software making the following assumptions: a two-tailed significance level α = 0.05, power 80%, and effect size *d* = 0.3 the sample size was estimated to be 280 patients. The study population was comprised of patients with a diagnosis of ESRD and undergoing hemodialysis referred to Shahrvand Dialysis in Sari. A convenience sampling method was used. Inclusion criteria were: ability to read and write, age 18 years and older. Exclusion criteria were: alcoholism, mental, emotional and verbal problems, decreased level of consciousness, gastrointestinal diseases such as peptic ulcer and gastroesophageal reflux disease, and congestive heart failure.

### Instruments

Data collection tools included a demographic registration form and Littleton's Body Image Concern Inventory Questionnaire (BICI) and the Assessment of Self-care Behaviors with Arteriovenous Fistula (ASBHD-AVF).

#### Body Image Concern Questionnaire

The 19-item Body Image Concern Questionnaire was first designed and validated by Littleton et al. (26). This questionnaire examines a patient 's dissatisfaction, fear and embarrassment regarding appearance, checking and hiding perceived imperfections, and the interference of a patient 's fear of appearance with social performance. The response options were a five-point Likert scale ranging from never = 1 to always = 5 (26). The total score of the questionnaire can varys between 19 and 95, and the highest score indicates more dissatisfaction with the body image or appearance. The reliability of this questionnaire in a sample of university students using Cronbach's alpha method was 0.93, the correlation of total items was between 0.32 and 0.72 and finally the validity coefficient of this questionnaire was 0.83 (26). The BICI has been translated into Persian and validated with satisfactory psychometric properties in an Iranian population (27–29). In the present study, the reliability of the questionnaire by Cronbach's alpha method was 0.94.

#### Assessment of Self-Care Behaviors With Arteriovenous Fistula

ASBHD-AVF consists of 16 items in two dimensions; that include the management of signs and symptoms (6 items) and the prevention of complications (10 items). The total score is between 16 and 80, and the highest score indicates a higher level of caring behavior (30). Several Persian studies have used ASBHD-AVF and it has reported high validated with satisfactory psychometric properties among Iranian populations with hemodialysis patients (31). The reliability of the questionnaire was calculated using Cronbach's alpha and was equal to 0.87.

### Ethical Considerations

Approval from the Ethics Committee at the Mazandaran University of Medical Sciences was obtained (Study ID number IR.MAZUMS.REC.1398.611). Written informed consent was obtained from all patients. The researchers went to the Shaharvand Dialysis and explained the objectives of the study to those who wanted to participate in the study.

### Data Analysis

Data were analyzed using SPSS26. The normal distribution of continuous quantitative data was investigated using the Kolmogorov-Smirnov test. Simple linear regression was used to analyze the relationship between self-care variables and body image in hemodialysis patients. Moreover, variables that were significant in simple linear regression were tested simultaneously in multiple linear regression. The significance level of all tests was considered less than α < 0.05.

## Results

### Sample Characteristics

The subject's age was mean = 56.97; SD = 13.48 (age range 21–94 years), respectively (Table 1) (Of these, 90.4% were married, 56.1% were men, and 60.4% had moderate economic status and about 86.1% of these patients had a diploma. Total score of self-care behavior (95%: CI: 64.44, 67.21, SD = 11.63, Mean = 65.82) and body image concern (CI 95%: 29.19–32.40, SD = 13.48, Mean = 30.80).

**Table 1:** Demographic characteristics of the patients in the study (*n* = 280).

	**Variable**	**Frequency (percentage)**
Sex	Male	157 (56.1%)
	Female	123 (43.9%)
Marital status	Single/non-partnered	17 (6.1%)
	Married	253 (90.4%)
	Divorced	9 (3.2%)
	Widowed	1 (.4%)
Economic status	Lower income	77 (27.5%)
	Middle income	169 (60.4%)
	Middle-high income	32 (11.4%)
	High income	2 (.7%)
Level of education	Under high school diploma	241 (86.1%)
	Bachelor and master	32 (11.4%)
	Higher than a master's degree	7 (2.5%)
Age		**Mean** **±standard deviation (S.D)**
		56.98 ± 13.48

Table 2 shows the results of a simple linear regression between the variables of age, sex, level of education and self-care behavior with concern about body image, age variables (*B* = −0.164, β = −0.163, *P* = 0.007), gender (*B* = 4.169, β = 0.154, *p* = 0.011) and self-care behavior (*B* = −0.162, β = −0.140, *p* = 0.020) are predictable. However, there was no significant relationship between education level variables (*B* = 0.597, β = 0.019, *p* = 758) economic status (*B* = 1.42, β = 0.066, *p* = 0.247) and body image concerns. Table 2 provides the results of a multiple linear regression, it was determined that after entering the age variables (*B* = −0.179, β = −0.179, *p* = 0.003), gender (*B* = 4.072, β = 0.150, *p* = 0.011) and self-care behavior (*B* = −0.190, β = −0.164, *p* = 0.006). Multiple regression model became significant. This regression model explains 7.5% of the predictors of body image concern in hemodialysis patients.

**Table 2:** Results of body image predictor variables (*n* = 280).

**Predictive variables**	**Simple linear regression**				**Multiple linear regression**			
	***B***	**β**	***p*-value**	**CI 95%**	***B***	**β**	***p*-value**	**CI 95%**
Age (year)	−0.164	−0.163	0.007	−0.282 to 0.042	−0.179	−0.179	0.003	−0.296 to −0.063
Gender (man/woman)	4.169	0.154	0.011	0.978 to 7.36	4.072	0.15	0.011	0.948 to 7.197
Level of education	0.597	0.019	0.758	−3.120 to 4.277	-	-	-	-
Economic status	1.42	0.066	0.247	−1.124 to 3.983	-	-	-	-
Self-care behavior	−0.162	−0.14	0.020	−0.299 to −0.025	−0.19	−0.164	0.006	−0.324 to −0.055

## Discussion

In this study, variables such as age, sex and self-care behavior were found to predict body image concerns in patients with hemodialysis. However, no meaningful relationships were found between variables such as education level and economic status with body image concerns. A study by Chen (10) entitled Body Image and Related Factors among Cervical Cancer Patients in China who have had surgical treatment showed that in women, there are fewer active coping strategies and people with higher education are more likely to have a lower body image (10). Body image consists of perceptions, thoughts, feelings, and behaviors related to the appearance, abilities, and functions of the body (32). Concern of body image is determined by sociocultural ideals and also sociocultural norms as shown in the media, friends, and peers make people internalize certain beliefs and ideas about ideal body shape (33). The triple influence model also states the important effects of sociocultural norms on body image (34).

The results of this study are consistent with the present study in terms of gender and may indicate that women undergoing hemodialysis may be more concerned about body image than men. In addition, the results of the present study are consistent with the results by Sadeghian et al. The results of a study by Sadeghian et al. indicates that hemodialysis patients can experience positive and negative psychological changes. In addition, the ability to take care of self predicts moderate psychological changes. Because it has had a positive and significant relationship with post-traumatic growth and a negative and significant relationship with morale (6).

Self-care behavior refers to the practice of activities that patients initiate and do to keep a healthy life. Self-care behavior is a careful action when it is done effectively. Self-care behavior is the emphasis of managing or solving the problems involved with the external factors under the traditions and cultures (35). Self-care behavior among hemodialysis patients refers to activities that promote survival, functional integration, and well-being (36). As a result, one can say that there can be a relationship between self-care behavior and body image concern, and by promoting self-care, body image anxiety can be reduced in patients undergoing hemodialysis. Wu's et al. (37) study, that investigated the dynamic changes in body image and quality of life in patients with breast cancer, has shown that body image predicts the quality of life of patients with breast cancer. Dynamic changes in body image and quality of life will be useful for combined surgical decisions in patients with breast cancer (37).

This study is consistent with current research and it can be concluded that there may be a relationship between body image changes and self-care. Moreover, the current study is in line with Pakzad et al. (38) study, that aims to investigate the effect of a self-care program based on modeling theory and role modeling on body image development of patients with colorectal cancer. Pakzad's study suggests that the use of a self-care program based on modeling theory and role model can play a key role in developing body image of patients with colon cancer (38). The results of Pakzad's study, along with the current study, suggest that there may be a relationship between self-care behavior and body image. Cook's research, which offers a model to understand the positive role of body image in the treatment of eating disorders, suggests that adaptation and self-care in the mind are considered as potential goals of occupational therapy in fostering a positive body image among people with irregular eating behaviors (15).

Self-care behavior has been related with clinical consequences in chronic kidney disease, and suitable self-care behavior may reduce adverse outcomes (39). Self-care behavior allows patient s to have additional control over their chronic disease and the monitoring of their health (40). Their findings are consistent with the present study, meaning that body image in patients with hemodialysis and self-care behavior are interrelated. The results of studies conducted by Poorgholami et al., is consistent with our study, and shows that information about the self-care program has a positive effect on increasing self-esteem in patients undergoing hemodialysis (41).

Consequently, it can be concluded that improving the level of self-care can reduce the concern about body image in patients with hemodialysis. Previous studies of Iranian patients undergoing hemodialysis have often dealt with the physical and psychological complications associated with dialysis. However, our goal was to study the relationship between self-care behavior and body image problems in hemodialysis patients in Iran. This study contained limitations, including non-random sampling and population in a given area of the country; therefore, it is suggested that further studies be undertaken in other parts of the country with diverse ethnic backgrounds.

## Conclusion

The findings of the present study identified that the older patients undergoing hemodialysis are less concern about their body image. Also, with increasing levels of self-care behaviors in patients with chronic renal failure who undergo hemodialysis there was less concern about body image. However, in woman body image was a strong predictor.

## Recommendation

This study suggests that, by increasing the awareness of nurses in recognizing the factors affecting the mental image of patients undergoing hemodialysis, the concerns of patients can be reduced to some extent. This in turn can lead to improved self-care behaviors and thereby reducing multiple referrals of patients to medical centers and ultimately lead to lower costs.

## Data Availability Statement

The raw data supporting the conclusions of this article will be made available by the authors, without undue reservation.

## Ethics Statement

The studies involving human participants were reviewed and approved by IR.MAZUMS.REC.1398.611. The patients/participants provided their written informed consent to participate in this study.

## Author Contributions

All authors have participated in conception and design, or analysis and interpretation of the data, drafting the article or revising it critically for important intellectual content, and approval of the final version.

## Conflict of Interest

The authors declare that the research was conducted in the absence of any commercial or financial relationships that could be construed as a potential conflict of interest.

## Publisher's Note

All claims expressed in this article are solely those of the authors and do not necessarily represent those of their affiliated organizations, or those of the publisher, the editors and the reviewers. Any product that may be evaluated in this article, or claim that may be made by its manufacturer, is not guaranteed or endorsed by the publisher.
